# Cardiovascular complications in COVID‐19 patients with or without diabetes mellitus

**DOI:** 10.1002/edm2.218

**Published:** 2020-12-25

**Authors:** Temidayo Abe, Obiora Egbuche, Joseph Igwe, Opeyemi Jegede, Bivek Wagle, Titilope Olanipekun, Anekwe Onwuanyi

**Affiliations:** ^1^ Internal Medicine Residency Program Morehouse School of Medicine Atlanta GA USA; ^2^ Department of Cardiovascular Disease Morehouse School of Medicine Atlanta GA USA; ^3^ Department of Epidemiology and Biostatistics University of North Texas Health Science Center Fort Worth TX USA; ^4^ Department of Medicine Morehouse School of Medicine Atlanta GA USA; ^5^ Department of Hospital Medicine Covenant Heart System Knoxville TN USA

**Keywords:** cardiovascular complications, COVID‐19, diabetes mellitus

## Abstract

**Introduction:**

Coronavirus disease 2019 (COVID‐19) has become a major global crisis. Preliminary reports have, in general, indicated worse outcomes in diabetes mellitus (DM) patients, but the magnitude of cardiovascular (CV) complications in this subgroup has not been elucidated.

**Methods:**

We included 142 patients admitted with laboratory‐confirmed COVID‐19 from April 1st to May 30th 2020; 71 (50%) had DM. We compared baseline demographics and study outcomes between those with or without DM using descriptive statistics. Multivariate logistic regression was used to estimate the adjusted odds ratio for the study outcomes in DM patients, compared to those without DM, stratified by age, sex and glycaemic control. CV outcomes of interest include acute myocarditis, acute heart failure, acute myocardial infarction, new‐onset atrial fibrillation and composite cardiovascular end‐point consisting of all individual outcomes above.

**Result:**

Mean age was 58 years. The unadjusted rates were higher in DM patients compared to non‐diabetics for the composite cardiovascular end‐point (73.2% vs. 40.6% *p* < .0001), acute myocarditis (36.6% vs. 15.5% *p* = .004), acute heart failure (25.3% vs. 5.6% *p* = .001), acute myocardial infarction (9.9% vs. 1.4% *p* = .03) and new‐onset atrial fibrillation (12.7% vs. 1.4% *p* = .009). After controlling for relevant confounding variables, diabetic patients had higher odds of composite cardiovascular end‐point, acute heart failure and new‐onset atrial fibrillation.

## Graphical abstract

In this study, we investigated cardiovascular complications in 142 COVID‐19 patients with versus without diabetes mellitus. Patients with diabetes mellitus were more likely to be older, have more comorbidities, and less likely to receive life‐saving medications. After adjusting for relevant comorbidities, the adjusted odds for composite cardiovascular endpoint, acute heart failure, and new onset atrial fibrillation were significantly higher among patients with diabetes mellitus.

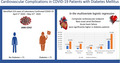

## 1 INTRODUCTION

Coronavirus disease 2019 (COVID‐19) has become a major global crisis. Although initially thought to affect only the respiratory system, recent studies have demonstrated gastrointestinal, neurological and cardiovascular sequelae.^1^ However, patients with underlying cardiovascular disease (CVD) tend to have worse outcomes compared to those without CVD.^1^ Also, COVID‐19 is associated with high rates of cardiovascular complications such as acute myocarditis (up to 28%), acute heart failure (23%) and arrhythmias (17%).^1^, ^2^ Finally, those who develop cardiovascular complications like acute myocarditis and acute heart failure are more likely to die.^1^, ^2^ Diabetes mellitus (DM) represents a significant health burden in the United States and is associated with severe illness in patients with COVID‐19.^3^ While preliminary reports have, in general, indicated worse outcomes in diabetics, the magnitude of CVD complications in this subgroup has not been fully elucidated.

## 2 METHODS

**FIGURE 1:**
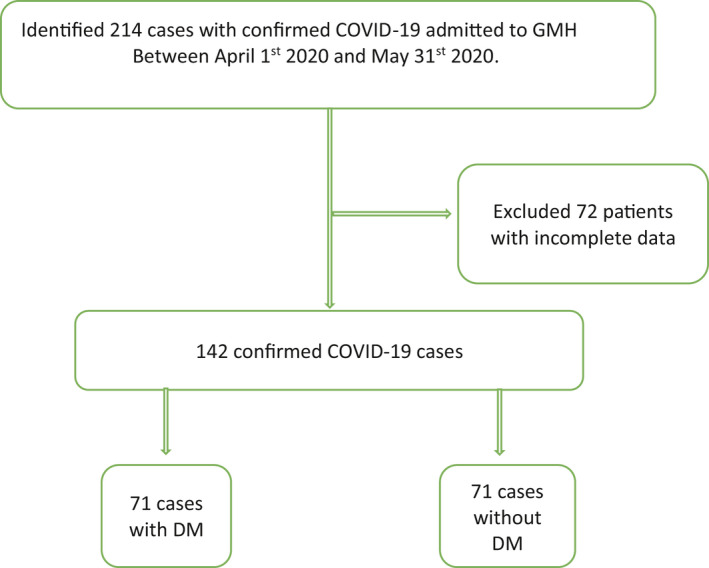
Flow chart of our study design. COVID‐19 represents coronavirus disease 2019, DM, diabetes mellitus; GMH, Grady Memorial Hospital

This study was conducted at Grady Memorial Hospital, the largest academic centre in Georgia, United States. This study was approved by our institutional review board with a health insurance portability and accountability act (HIPAA) waiver due to minimal risk to the privacy of individuals. We queried the hospital's electronic medical records from April 1st to May 30th 2020, to identify patients with laboratory‐confirmed COVID‐19 (Figure 1).

We collected patients level data, including demographics, comorbidities and in‐hospital complications. This study's primary end‐point is to investigate DM’s impact on acute heart failure, acute myocarditis, acute myocardial infarction, new‐onset atrial fibrillation and composite cardiovascular end‐point among COVID‐19 patients. Acute heart failure was defined as the presence of clinical signs and symptoms of heart failure, combined with radiologic and laboratory evidence, acute myocarditis as troponin I greater than 0.03 ng/ml, acute myocardial infarction according to the fourth universal definition of myocardial infarction,^4^ new‐onset atrial fibrillation based on electrocardiographic changes and composite cardiovascular end‐point consisting of all individual outcome above.

Stata MP V16 was used for statistical analysis. We compared baseline characteristics between patients with DM and those without DM. For categorical variables, differences between demographic, clinical and biochemical characteristics between the two groups were assessed using the chi‐squared test. In contrast, an independent Students *t*‐test was used for continuous variables.

We compared the incidence of acute heart failure, acute myocarditis, acute myocardial infarction, new‐onset atrial fibrillation and composite cardiovascular end‐point. Descriptive statistics were reported in frequencies with percentages for categorical variables, while continuous variables were reports in mean, standard deviation, median, and 25th and 75th percentiles. Multivariate logistic regression was used to estimate the adjusted odds ratio for the study outcomes in patients with DM, compared to those without DM. Composite cardiovascular end‐point was stratified by age, sex and glycaemic control (controlled (A1c < 7) versus uncontrolled (A1c > 7)).

## 3 RESULTS

We included a total of 141 patients with confirmed COVID‐19, of which 71 (50%) had DM. Among patients with DM, 19 (27%) had controlled DM, and 52 (73%) had uncontrolled DM. The mean age of patients with DM was lower compared to those without DM. Patients with DM had higher rates of comorbidities, including obesity, dyslipidaemia and hypertension (Table 1).

**TABLE 1:** Baseline Characteristic for Study Cohort

COVID−19 Confirmed			
	No DM (*n* = 71)	DM (*n* = 71)	*p*‐Value
Age (years), mean	60	56	<.0001
Gender (Frequency)			
Male	52.8	47.2	.39
Female	45.3	54.7	
Race			
African American	73.2	81.7	.23
White	9.9	7.0	.54
Asian	2.8	0.00	.15
Native American	0.00	0.00	.00
Hispanic	11.3	7.0	.52
Other	2.8	4.2	.64
Health Insurance	73.2	66.2	.36
Comorbidities			
Alcohol abuse	35.2	14.1	.003
Tobacco abuse	27.6	22.7	.53
COPD	14.0	5.6	.09
Asthma	5.6	5.6	1.0
Dyslipidaemia	28.2	49.3	.01
Valvular Heart Disease	5.6	5.6	1.0
Chronic Kidney Disease	14.1	26.7	.06
Obesity	42.3	59.5	.04
Congestive Heart Failure	12.7	21.1	.17
Chronic Liver Disease	9.9	0.0	.007
Hypertension	60.6	84.5	<.0001
Prior Stroke	22.5	15.5	.28
Alcohol Abuse	25.9	25.4	.95
Pulmonary Hypertension	1.4	0.0	.35
Coronary Artery Disease	14.1	12.7	.42
Peripheral Artery Disease	7.0	12.7	.26
Haemodialysis	5.6	7.0	.73
HIV	8.5	2.8	.15
History of Atrial fibrillation	8.5	14.1	.28
In‐hospital management			
Steriods	10.0	14.1	.4
Hydroxloroquine	38.0	18.3	.009
Remdesivir	25.4	9.9	.02
Complications			
DVT	5.6	9.9	.34
Pulmonary Embolism	2.8	9.9	.09
Acute Kidney Injury	28.2	42.3	.08
Acute Kidney Injury w RRT	2.8	11.3	.05
Acute Respiratory Failure	22.5	52.1	<.0001
Death	8.5	23.9	.46

Abbreviations: COPD, chronic obstructive pulmonary disease; DM, diabetes mellitus; DVT, deep vein thrombosis; HIV, human immunodeficiency virus; w RRT, with renal replacement therapy.

The unadjusted rates were higher in DM patients compared to non‐diabetics for the composite cardiovascular end‐point (73.2% vs. 40.6% *p* < .0001), acute myocarditis (36.6% vs. 15.5% *p* = .004), acute heart failure (25.3% vs. 5.6% *p* = .001), acute myocardial infarction (9.9% vs. 1.4% *p* = .03) and new‐onset atrial fibrillation (12.7% vs. 1.4% *p* = .009). After controlling for relevant confounding variables, diabetic patients had higher odds of composite cardiovascular end‐point, acute heart failure and new‐onset atrial fibrillation. In the stratified analysis, a nonsignificant higher odds for composite cardiovascular end‐point was found among older patients, male patients and those with uncontrolled DM (Tables 2 and 3).

**TABLE 2:** Association between DM and cardiovascular outcomes among hospitalized patients with COVID‐19

COVID−19						
	No DM(%)	DM (%)	aOR	Lower CI	Upper CI	*p*‐Value
Acute Myocarditis	15.5	36.6	2.0	0.7	5.6	.2
Acute Heart Failure	5.6	25.3	7.9	1.6	40.3	.01
Acute MI	1.4	9.9	7.7	0.2	315.7	.3
New‐onset AF	1.4	12.7	28.7	1.3	647.9	.04
Composite CVD end‐point	40.6	73.2	3.0	1.0	8.9	.04

Abbreviations: AF, atrial fibrillation; aOR, adjusted odds ratio; CI, confidence interval; CVD, cardiovascular; DM, diabetes mellitus.

**TABLE 3:** Association between DM and composite cardiovascular end‐point among hospitalized patients with COVID‐19, stratified by age, sex and diabetes status

	95% CI			
	OR	Lower	Upper	*p*‐Value
Age (years)				
18–50	REF	REF	REF	REF
50	2.2	0.5	9.6	.2
Gender				
Female	REF	REF	REF	REF
Male	1.6	0.4	25.4	.2
Diabetes Status				
Controlled (A1C ≤ 7%)	REF	REF	REF	REF
Uncontrolled (A1C > 7%)	1.5	0.2	9.7	.6

Abbreviations: A1C, haemoglobin A1C; AF, atrial fibrillation; aOR, adjusted odds ratio; CI, confidence interval; DM, diabetes mellitus; REF, reference group.

## 4 DISCUSSION

In this study, DM was associated with worse cardiovascular outcomes, including composite cardiovascular end‐point, acute heart failure and new‐onset atrial fibrillation. Cardiovascular complications in COVID‐19 is thought to be related to direct myocardial injury, microvascular damage, cellular hypoxia and cytokine release.^5^ The role of innate immune system dysregulation in severe COVID‐19 disease in DM patients has yet to be well defined. However, DM patients have been demonstrated to exhibit severe immune response when infected with COVID‐19.^6^ We speculate that the inflammatory response associated with DM could explain the worse cardiovascular outcomes, among other possible explanations such as increased comorbidities and decreased utilization of life‐saving medications, as demonstrated in this study (Table 1). Further research is needed to understand the disease process's pathophysiologic mechanisms in diabetics and to devise treatment strategies to mitigate complications. The study must be interpreted with caution as over 80% of our study population were African Americans, limiting our study results’ generalizability. Also, the study population consisted predominantly of patients with uncontrolled DM, which might impact the study results.

## CONFLICT OF INTEREST

Author and co‐authors have no conflict of interest.

## AUTHOR CONTRIBUTION

Abe Temidayo and Obiora Egbuche were involved in study conception, data acquisition, interpretation of results, and manuscript writing. Joseph Igwe, Opeyemi Jegede, Bivek Wagle were involved in data acquisition and statistical analysis. Titilope Olanipekun and Anekwe Onwuanyi were engaged in the critical review.

## Data Availability

The data that support this study's findings are available on request from the corresponding author [T.A]. The data are not publicly available due to them containing sensitive information that could compromise participant privacy.
